# Separation of the Lipid Fraction from Cocoa Bean Husks Using Ethyl Acetate as Solvent in Ultrasound-Assisted Process

**DOI:** 10.3390/foods15132275

**Published:** 2026-06-25

**Authors:** Lauana Fernandes Silva, Stenio Cristaldo Heck, Vitor Augusto dos Santos Garcia, Camila da Silva

**Affiliations:** 1 Programa de Pós-Graduação em Engenharia Química, Departamento de Engenharia Química, Universidade Estadual de Maringá, Av. Colombo 5790, Maringa 87020-900, PR, Brazil; 2 Departamento de Tecnologia, Centro de Tecnologia, Universidade Estadual de Maringá, Av. Ângelo Moreira da Fonseca 1800, Umuarama 87506-370, PR, Brazil; stenioheck@gmail.com; 3 Departamento de Produção Vegetal, Faculdade de Ciências Agronômicas, Universidade Estadual Paulista “Júlio de Mesquita Filho”, Botucatu 18610-034, SP, Brazil

**Keywords:** *Theobroma cacao* L., ultrasound-assisted extraction, waste recovery, phytosterols, fatty acids

## Abstract

This study aimed to obtain the lipid fraction from cocoa bean husks by applying ethyl acetate as an extraction solvent in an ultrasound-assisted extraction process. The effects of temperature (T), time (t), and solvent:husk ratio (R) on the lipid fraction (LF) yield were evaluated. The removal of minor compounds (phytosterols and tocopherols) and total phenolics was evaluated under selected conditions, as well as the value of conjugated dienes (CDs). Extraction with n-hexane was performed for comparative purposes. The prediction of the solubility of the main compounds identified in the solvents used was conducted. The influence of the variables on LF removal was T > t > R, which provided the highest result (13.54 ± 0.47 wt%) at the highest levels adopted (70 °C, 60 min, 12 g/mL), a value 23% higher than that obtained using n-hexane. Under these conditions, there is also greater recovery of minor compounds from the peels, especially β-sitosterol, which was quantified at 43 to 50% of the concentration of these compounds. The use of ethyl acetate provided greater removal of minor compounds and total phenolics, resulting in lower primary lipid oxidation products (CD value). The relationship between these properties was evidenced by the Pearson correlation matrix, especially for stigmasterol, campesterol, total phenolics, and total minor compounds. The thermodynamic modeling reveals regimes ranging from full miscibility of liquid solutes to limited solubility of phytosterols and gallic acid; however, the contrast with experimental data indicates that real extraction is limited by kinetic barriers and plant matrix effects. The solvent extractor did not influence the fatty acid profile of the LF obtained, consisting mainly of saturated fatty acids (palmitic and stearic).

## 1. Introduction

Cocoa seeds, also called cocoa beans, consist of an outer shell that encloses two cotyledons and a small germ [1]. These husks represent one of the main byproducts of the cocoa industry, corresponding to ~10 to 17% of the total weight of the bean, and are composed mainly of dietary fiber, phenolic compounds, and a variable lipid fraction, generally between 5 and 18 wt% [2,3,4].

Enhancing the value of this byproduct involves strategies for its breakdown into products with higher added value, which aim to increase the portfolio of products derived from cocoa. One area of interest is the removal of lipids, which results in a product for direct commercialization and defatted husks that have a high content of phytochemicals in their composition. In a circular economy approach, extraction methods can be applied to recover compounds from defatted husks, primarily targeting the recovery of the phenolic fraction in this material, thus adding even more value to the byproduct.

Stearic and palmitic acids are the predominant fatty acids in the lipids obtained from cocoa bean husks, with 23.7–32% and 26.5–38.5%, respectively [4,5,6]. The low presence of polyunsaturated fatty acids in the lipid fraction, generally <5% [5], is associated with the high oxidative stability. Okiyama et al. [2] reported the tocopherol composition, highlighting (γ + β)-tocopherol, which represents ~60% of the total of these compounds identified, and Disca et al. [5] showed a predominance of β-sitosterol in the phytosterol composition.

To establish a process for obtaining the lipid fraction from a given matrix, one of the first aspects to consider involves the choice of extraction solvent. Ethyl acetate has been little explored for the lipid extraction from cocoa husks; however, this solvent is food-grade and classified by the Food and Drug Administration (FDA) as Generally Recognized As Safe (GRAS), standing out for its low toxicity, biodegradability, and ease of recovery [7]. Stevanato et al. [8] reported the effectiveness of ethyl acetate for removing the lipid fraction from oilseed seeds with yields close to that of n-hexane and greater selectivity in relation to the removal of phytosterols and tocopherols. Additionally, Kalkan and Maskan [9] found that the energy consumption during oil extraction with this solvent is significantly lower than that of n-hexane, and attribute this result to the fact that ethyl acetate has a lower vapor pressure, thereby exhibiting higher energy efficiency and oil recovery.

The use of ethyl acetate in an ultrasound-assisted extraction (UAE) process to obtain lipids from cocoa husks has not been investigated, which motivated the development of this research, with the hypothesis that this strategy will be effective in obtaining the lipid fraction of the matrix in question with yield and quality comparable to other techniques. The characteristics that distinguish the UAE are widely presented in the literature and involve its classification as an intensification method since high yields are obtained in short extraction times compared to conventional techniques [10,11]. The application of ultrasound is capable of generating a porous structure in the cell wall, thus facilitating the penetration of solvent and the release of oil [12]. The effectiveness of UAE is influenced by the extraction temperature and solvent availability, since these variables influence the formation of cavitation bubbles in the extraction medium [9,11]; therefore, the evaluation of the effect of these variables is commonly verified in the application of this technique [13,14].

Therefore, the objective of this work was to evaluate the extraction of the lipid fraction from cocoa bean husks using ethyl acetate as a solvent. For this purpose, ultrasound-assisted extraction was employed, evaluating the effect of temperature, extraction time, and solvent/husks ratio on the lipid fraction yield. Extraction using n-hexane was also performed for comparison purposes. The efficiency of recovery of minor compounds and total phenolic compounds into the lipid fraction was evaluated, as well as the formation of conjugated dienes. The lipids were further characterized in terms of fatty acid composition. The COSMO-SAC (Conductor-like Screening Model for Segmented Activity Coefficients) model was applied to predict the solubility of the main compounds identified in ethyl acetate and n-hexane. This study is part of a broader project that aims to further evaluate the removal of compounds from defatted husks using different extraction methods and solvents. It also aims to assess the applicability of the compounds obtained and the residual solid biomass in order to achieve full utilization.

## 2. Materials and Methods

### 2.1. Raw Material: Origin and Preparation

Cocoa bean husks (6.61 ± 0.24 wt% moisture) obtained from fermented and roasted beans of the Trinitário variety, supplied by Fazenda Bate Corrente, Espírito Santo, Brazil (20°54′58.1″ S; 41°11′38.5″ W) were used. The husks were ground in an electric grinder (Philco, 053901040 (Philco, Manaus, AM, Brazil)) and subsequently classified according to particle size using sieves (Bertel/ASTM). The fraction that passed through the 20-mesh sieve and remained on the 28-mesh sieve (average diameter of 0.715 mm) was used to conduct the experiments.

### 2.2. Chemical Products

Ethyl acetate (99.5%, Exodus Scientific, Sumaré, SP, Brazil) and n-hexane (99.0%, Synth, Diadema, SP, Brazil) were used as extraction solvents.

Heptane (99.0%, Synth, Diadema, SP, Brazil), N,O-bis(trimethylsilyl)trifluoroacetamide containing 1% trimethylchlorosilane (BSTFA/TMCS) (Sigma-Aldrich, St. Louis, MO, USA), and 5α-cholestane (Sigma-Aldrich, St. Louis, MO, USA) were used for the analysis of minor compounds. Folin–Ciocalteu reagent (Dynamic, Indaiatuba, SP, Brazil) and sodium carbonate (Anidrol, Diadema, SP, Brazil) were used for the determination of total phenolic compounds in the lipid fraction. For conjugated dienes determination, n-hexane (99.0%, Synth, Diadema, SP, Brazil) was used as solvent. Methanol (99.9%, Panreac, Barcelona, Spain), potassium hydroxide (56.11%, Dynamic, Indaiatuba, SP, Brazil), and sulfuric acid (>95%, Anidrol, Diadema, SP, Brazil) were used for methylation in fatty acid analysis.

### 2.3. Obtaining the Lipid Fraction

The extraction was conducted in an ultrasonic bath (Ultronique, Q 5.9/40 A, ultrasonic frequency of 25 kHz) with indirect contact, using maximum power (165 W) [14]. The two-stage procedure was adopted after conducting preliminary tests (Table S1, Supplementary Materials) that indicated greater recovery of the lipid fraction using this procedure. The efficiency of using ethyl acetate compared to ethanol was also verified and presented in this table.

The procedure for obtaining the lipid fraction consisted of weighing 3.5 g of husks (on a dry basis) into a 250 mL Erlenmeyer flask, followed by the addition of the extraction solvent (as indicated by the experimental condition). The flask was then placed in the center of an ultrasonic bath, stabilized at the test temperature, coupled to a condenser connected to a water recirculation bath at 15 °C (Marconi, MA 184, Piracicaba, SP, Brazil). After the first extraction stage, the extraction medium was filtered, and the partially defatted husks retained on the filter were dried at 80 °C for 1 h (Marconi MA035, Piracicaba, SP, Brazil). The mass of husks obtained was recorded (for calculating the solvent volume), and these were used in the second extraction stage, following the same procedure adopted in the first stage. Finally, the filtrates were combined, and solvent removal was conducted in a rotary evaporator (Marconi, MA 120, Piracicaba, SP, Brazil) at 60 °C and −0.8 bar. The remaining solvent was removed in a circulating air oven at 80 °C until a constant weight was obtained, resulting in the lipid fraction (consisting of lipids and polar/nonpolar compounds). The solvent volume in each stage was calculated considering the mass of husks on a dry basis.

The extractions were conducted following an experimental design generated by Statistica^®^ 8.0 software (StatSoft, Inc., Tulsa, OK, USA), considering three independent variables tested at three levels each: temperature (40, 55 and 70 °C), extraction time (20, 40 and 60 min) and solvent/sample ratio (8, 10 and 12 g/mL). As a response variable, the lipid fraction yield (LFY) was considered, which was calculated based on the mass of extracted material obtained in relation to the initial mass of the husks used (on a dry basis). Statistica^®^ 8.0 software was used to evaluate the effect of the independent variables on the LFY (significant difference in *p* > 0.05).

Experiments were conducted using n-hexane as a solvent for comparative purposes, adopting the same two-step procedure, under the condition of maximum LFY obtained from the use of ethyl acetate.

### 2.4. Analytical Methods

The content of minor compounds in the lipid fraction was determined after derivatization of the sample with BSTFA/TMCS (1 mg/µL) at 60 °C for 30 min and addition of the internal standard (5α-cholestane). The analysis was conducted using gas chromatography–mass spectrometry (GC-MS) (Shimadzu, GC-MS-QP2010 SE, Tokyo, Japan) as described by Stevanato and Silva [15] (2019). For identification, the mass spectra of the compounds detected were compared with those of the NIST 14 library, the Pubchem database established by the National Library of Medicine, and the NIST standard reference database number 69 (NIST Chemistry WebBook). The results were expressed in mg of the compound per 100 g of husks, considering the compound content in the sample and LFY.

To determine the total phenolic content, the compounds were first extracted with methanol and water [16], and the compounds in the hydromethanolic extract were quantified using the Folin–Ciocalteu method [17] and a gallic acid standard curve. The results were expressed in mg of gallic acid equivalent (GAE) per 100 g of huks, considering the total phenolic content in the sample and LFY.

The oxidative state of the lipid fraction was evaluated by determining conjugated dienes using ultraviolet spectrophotometry [18]. For the analysis, 0.01 g of lipid fraction was dissolved in 10 mL of n-hexane, and absorbance was measured at 232 nm (Shimadzu UV-1900, Japan, Tokyo).

The fatty acid composition was determined after methylation of the lipid fraction, using a combination of an alkaline reaction (KOH/methanol, 2 mol/L) and an acidic reaction (5% H_2_SO_4_ in methanol). The sample was weighed (~60 mg) in a test tube with a cap, and 2 mL of alkaline solution was added. It was vortexed for 5 min at room temperature and heated in a water bath (100 °C) for 5 min. After cooling in an ice bath, 5 mL of acid solution was added, and the tube was placed in a water bath at 100 °C for 20 min. Cooling was performed, and 5 mL of heptane was added. After 24 h, the upper phase was removed and analyzed. The sample after methylation was analyzed under the chromatographic conditions indicated by Mello et al. [19], except for the heating ramp, which was defined as follows: initial temperature of 140 °C, heating up to 180 °C at 10 °C/min and then further heating up to 220 °C at 4 °C/min. Compounds were identified as described for minor compounds, and the percentage of each fatty acid was expressed as a percentage of normative area, considering the total area of the identified fatty acids.

The results of the analyses refer to averages of results obtained from extraction and analysis replicates, which were subjected to one-way ANOVA followed by Tukey’s post hoc (*p* ≤ 0.05), using Version 8 of Statistica software (StatSoft, Inc., Tulsa, OK, USA). Pearson correlation coefficients, ranges from −1 to 1, were obtained using PAST software (version 4.03) and used to establish the data correlation matrix.

### 2.5. Prediction of Compound Solubility Using COSMO-RS

The molecular geometries of the solutes and solvent were optimized, and the screening charges (σ-profiles) were calculated using the ORCA software (version 5.0.4) [20]. For all calculations, Density Functional Theory (DFT) was employed with the B3LYP functional [21,22] and the def2-TZVP basis set [23]. The polarization of the medium was modeled using the COSMO (Conductor-like Screening Model) module [24] of ORCA. The .orcacosmo output files generated by ORCA contain detailed information about the molecular surface, including the surface points and the screening charges ($q_{au}$) associated with each area ($a_{ang^{2}}$).

A MATLAB^®^ script was used to process these files, extracting the surface area ($a_{ang^{2}}$) and screening charge ($q_{au}$) or each point on the molecular surface. The screening charge density (σ) is then calculated as $\sigma =q_{au}/a_{ang^{2}}$. From these data, a screening charge density distribution profile (σ-profile) is constructed, representing the charge distribution on the molecular surface. This profile is essential for calculating the activity coefficients in the COSMO-SAC model.

The activity coefficients ($\gamma_{i}$) were obtained using a MatLab^®^ R2025b script using a COSMO-SAC formalism [25], parameterized from the σ profiles. This model considers contributions from electrostatic interactions, hydrogen bonds and dispersion forces, allowing the description of the non-ideal behavior of solutions.

For solid solutes, the free energy of fusion ($\Delta G_{fus}$) t a given temperature T is a critical factor for solubility. The Gibbs-Helmholtz equation can be used to relate $\Delta G_{fus}$ to the enthalpy of fusion ($\Delta H_{fus}$) and the melting temperature ($T_{m}$). The complete form includes the difference in heat capacity between the liquid and solid phases ($\Delta C_{p}$):

(1) $$\Delta G_{fus}(T)=\Delta H_{fus}(T_{m})\left(1-\frac{T}{T_{m}}\right)-\Delta C_{p}\left(T_{m}-T-Tln\left(\frac{T_{m}}{T}\right)\right)$$

where $\Delta H_{fus}$ is the enthalpy of fusion of the solute (kJ/mol); is the temperature of the system (K); *T_m_* is the melting point of the solute (K); $\Delta C_{p}=C_{p,líquido}-C_{p,sólido}$ is the difference in molar heat capacity between the liquid and solid phases (kJ/mol K).

Although Equation (1) is thermodynamically rigorous, obtaining precise $\Delta C_{p}$ values for the liquid and solid phases of phytosterols is challenging and often unavailable experimentally. The use of estimated $\Delta C_{p}$ values can introduce significant errors, leading to an underestimation of $\Delta G_{fus}$ and, consequently, an overestimation of solubility. or phytosterols, it was observed that the $\Delta C_{p}$ term in Equation (1) tended to cancel out a large part of the enthalpic contribution, resulting in unrealistically low $\Delta G_{fus}$ values and predictions of complete miscibility for solids. Therefore, the classical approximation was chosen, where $\Delta C_{p}\approx 0$, simplifying the equation to

(2) $$\Delta G_{fus}(T)=\Delta H_{fus}(T_{m})\left(1-\frac{T}{T_{m}}\right)$$

This approximation is widely accepted in the literature for predicting the solubility of organic solids [26] and has been shown to provide results more consistent with the expected physical behavior for the compounds studied. For liquid or miscible solutes, $\Delta G_{fus}$ is considered zero.

The mole fraction of the solute in the saturated phase ($X_{i}$) is calculated from the $\Delta G_{fus}$ and the activity coefficient ($\gamma_{i}$) of the solute in the solution, according to the modified Schröder-van Laar equation:

(3) $$\ln(X_{i})=-\frac{\Delta G_{fus}}{R\cdot T}-\ln(\gamma_{i})$$

where R is the ideal gas constant (8.314 × 10^−3^ kJ/mol K), and T is the temperature of the system (K).

The molar fraction (*X*_i_) must be physically limited to the interval [0, 1]. If the calculation of Equation (3) results in *X*_i_ > 1, the value is adjusted to *X*_i_ = 1, indicating complete miscibility of the solute in the solvent. Solubility in mol/L (S) is derived from the mole fraction (*X*_i_) and the molar volume of the mixture (*V_mix_*):

(4) $$S=\frac{X_{i}}{V_{mix}}$$

The molar volume of the mixture (*V_mix_*) is calculated as a weighted average of the molar volumes of the solute ($V_{m,soluto}$) and the solvent ($V_{m,solvent}$):

(5) $$V_{mix}=X_{i}\cdot V_{m,soluto}+(1-X_{i})\cdot V_{m,solvent}$$

Individual molar volumes are obtained from the molar mass (M) and density (ρ) of each component:

(6) $$V_{m,j}=\frac{M_{j}}{\rho_{j}\cdot 1000}$$

where *j* represents the solute or solvent, *M_j_* is the molar mass (g/mol), and $\rho_{j}$ is the density (g/mL). The factor of 1000 converts mL to L.

The use of *V_mix_* in Equation (4) is fundamental to obtaining solubility values in mol/L that are physically realistic, correcting the overestimation that would occur when using only the molar volume of the solvent.

The thermodynamic input data for the solubility calculations were carefully compiled and validated from reliable sources in the literature (Table 1) and databases, adopting a reference temperature of 298.15 K.

## 3. Results

### 3.1. Effect of Independent Variables on Lipid Fraction Yield

Table 2 presents the experimental conditions and the respective LFY obtained from cocoa bean husks. The values ranged from 7.12 wt% to 12.97 wt%, a variation of 82% of the values obtained.

Table 3, which presents the effects of the independent variables on the LPY, shows that the linear term of the independent variables considered was significant, with temperature (2.64) standing out, followed by time (2.32) and solvent/sample ratio (0.47). Regarding the interactions between the variables, statistical significance was found for the terms T × t, t × R and T × t × R.

**Figure 1:**
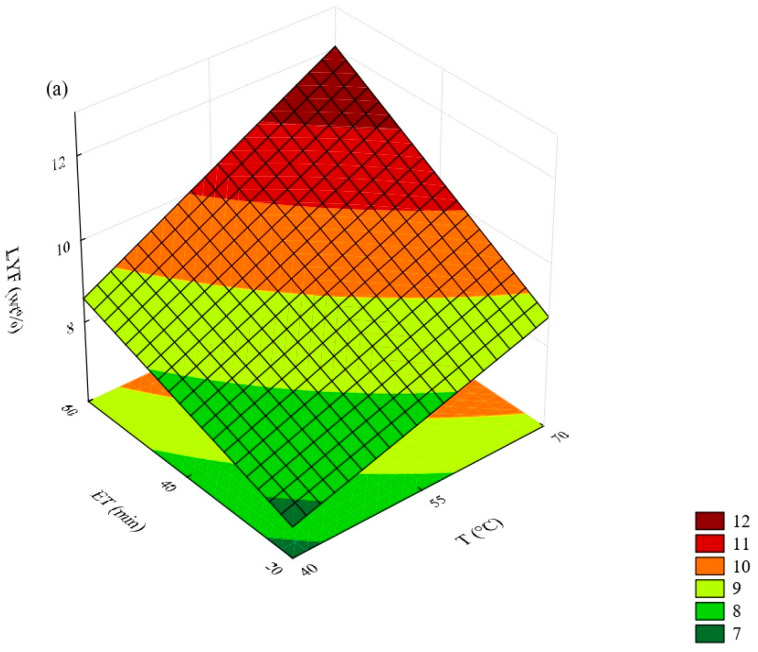

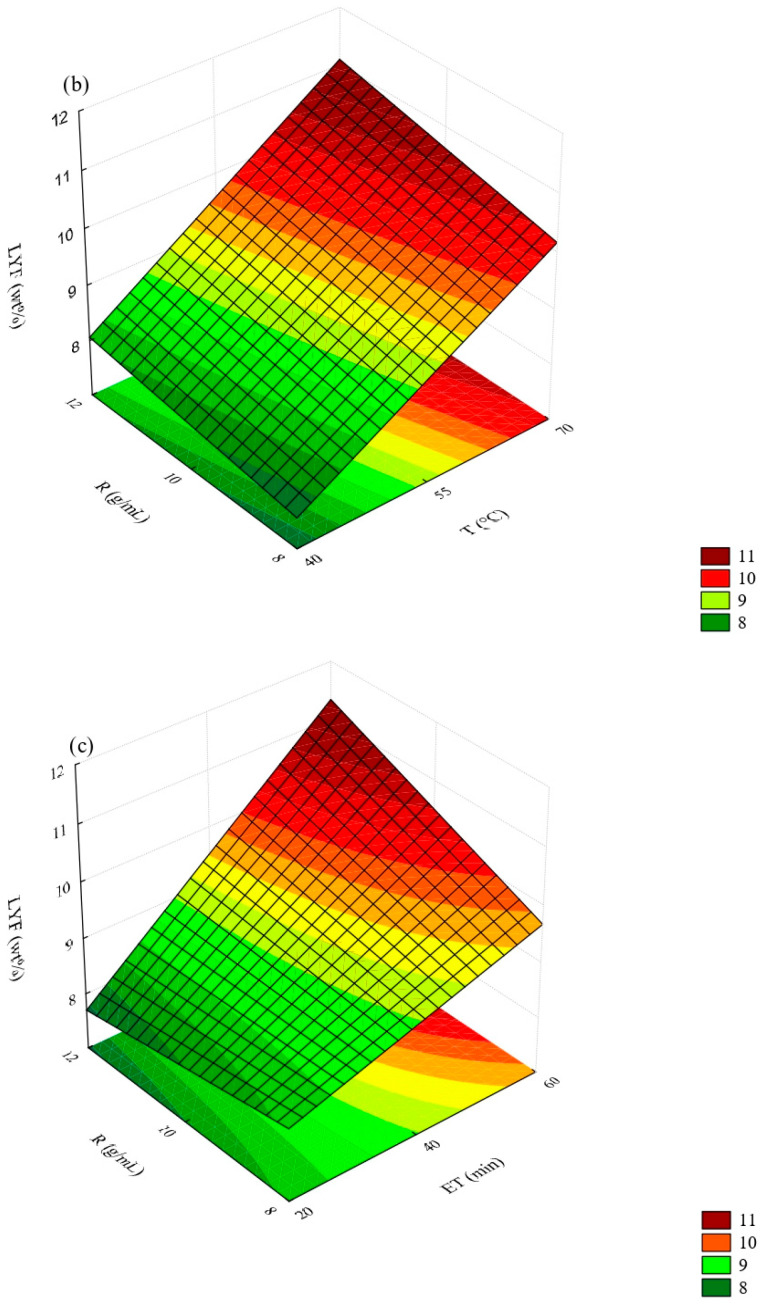
Response surface analysis for lipid fraction yield (LFY): T: temperature; ET: extraction time; R: solvent/sample ratio. Correlative effects of (**a**) ET and T, (**b**) R and T, and (**c**) R and ET. Each graph represents the function of two variables, keeping the third at the central point.

Figure 1 shows the response surfaces for the combinations between the independent variables, allowing visualization of the combined influence of temperature, time, and solvent/sample ratio on LYF.

In Figure 1a, it can be seen that thermal intensification favored the removal of lipids from the husks, reaching higher values at 70 °C, which showed a 38% increase compared to the extraction conducted at 40 °C (runs 4 and 8). This occurs due to the increased solubility of lipids and the reduction in solvent viscosity with increasing operating temperature, promoting greater diffusion of the lipid fraction into the extraction medium [31]. Kalkan and Maskan [9] explain that an increase in temperature causes the formation of more cavitation bubbles in the UAE, which explode near the membranes of plant cells, increasing shear stress and the area of interaction between the solvent and the cell membrane of the solid matrix. Ribas et al. [32] obtained higher diffusion coefficient values for rice bran oil with increasing temperatures from 25 to 55 °C using ethyl acetate, also showing that this increase made the extraction process more spontaneous and favorable, based on the negative values of the standard Gibbs energy variation.

Increasing the extraction time (Figure 1a,c) resulted in higher LPY values. It was observed that most of the lipid fraction was removed in 20 min, representing 84% (runs 1 and 3) and 65% (runs 6 and 8) of the value obtained in 60 min. This indicates that after 20 min of extraction, the most accessible compounds, which are present on the surface, are obtained, and after that, extraction is governed by diffusion, which involves the extraction of compounds located inside the matrix into the solvent medium. The term T × t (Table 2) was positive (*p* < 0.05), indicating that the partial disruption of cell walls favored by heating is more pronounced with increasing extraction time, resulting in greater lipid recovery.

From Figure 1a,b it is observed that LPY is higher with increasing solvent volume in the extraction medium; however, this variable showed less effect when compared to temperature and time (Table 3). The simultaneous increase in time and solvent availability promoted greater recovery of the lipid fraction; however, the effect of temperature acts independently of the amount of solvent in the extraction medium. The interaction T × t × R (0.74, *p* < 0.001) indicates that the effectiveness of the extraction process depends on the synergy between the three process variables. Thus, reducing T and R in order to minimize energy consumption leads to a reduction in the LPY, suggesting that longer extraction times need to be adopted.

Analysis of variance (ANOVA) was conducted (Table S2, Supplementary Materials) to establish the predictive equation (R^2^ of 0.93 and adjusted R^2^ of 0.86):

(7) LFY (wt%) = 8.96 + 2.62 T + 2.30 t + 0.45 R + 0.69 T t + 1.06 t R + 0.73 T t R

The F-test confirmed the significance of the model, since the calculated F-value (18.66) was higher than the tabulated F-value (3.58). From Equation (1), the condition for maximum LPY was determined to be 13.62 wt% (70 °C, 60 min, 12 g/mL), obtained from a desirability factor of 0.98 and corresponding to run 8 of the experimental design (Table 1). Conducting the experiment in quadruplicate under these conditions resulted in 13.54 ± 0.47 wt%, not differing significantly (*p* > 0.05) from the value predicted by Equation (1). Extraction conducted using n-hexane, for comparative purposes, resulted in an LPY of 10.94 ± 0.34 wt%.

The LPY obtained in this study with ethyl acetate proved to be superior to or comparable to the values reported in the recent literature for the extraction of the lipid fraction from cocoa bean husks. Okiyama et al. [2] achieved removal of ~90% of fat, which corresponds to a yield of 16.38 wt%, using pressurized ethanol. Thawornprasert and Somnuk [4], using n-hexane and ethanol, obtained 10.15 wt% and 8.50 wt% of lipids, respectively. Disca et al. [5] obtained yields of 5.0 to 8.0 wt% testing dichloromethane, petroleum ether, diethyl ether and n-hexane in Soxhlet extraction. Ridella et al. [33] report obtaining 7.23 wt% of fat by applying Soxhlet extraction with n-hexane. The difference between the results obtained with different solvents is correlated to the fact that ethanol and ethyl acetate remove lipids and other compounds such as phosphatides, polyphenols, pigments, sugars and soluble proteins [8]. Additionally, as a result of the two-step extraction conducted in this study, greater recovery of compounds soluble in ethyl acetate was obtained.

### 3.2. Extraction of Minor Compounds, Total Phenolics and Conjugated Diene Content

Table 4 presents the results of the removal of minor compounds and total phenolic compounds under different experimental conditions (selected to verify the influence of LPY and experimental conditions) and the content of conjugated dienes in the lipid fractions obtained.

From Table 4, it can be seen that ethyl acetate, under all conditions evaluated, provided greater removal of minor compounds, since n-hexane reached 72.22% and 42.29% of the lowest (run 2) and highest (run 8) values obtained with ethyl acetate. It can be inferred that n-hexane was tested under maximum LPY conditions with ethyl acetate and may present different results than when tested under other experimental conditions. However, Wei et al. [34] reported high solubility of β-sitosterol in ethyl acetate, due to the low polarity of this solvent, which reached a value 3.17 times higher compared to n-hexane at 50 °C.

Regarding the experimental conditions adopted with ethyl acetate, the greater availability of solvent at 70 °C and 60 min generally provided greater extraction of isolated minor compounds and consequently greater total removal of compounds. Ibrahim et al. [35] report obtaining fat from cocoa shell waste with a higher concentration of β-sitosterol by increasing the extraction temperature from 50 to 70 °C.

Among the minor compounds identified, β-sitosterol stands out, being predominant in all samples analyzed, representing 43 to 50% of the total composition. Previous studies report similar behavior for the phytosterol profile of cocoa husk fat [5,36].

Levels of (γ + β)-tocopherol were detected in the lipid fractions obtained with ethyl acetate, corresponding to 9.5 to 13.2% of the total minor compounds quantified. Disca et al. [5] also reported the presence of this tocopherol only in the lipid fractions of cocoa husks obtained by Soxhlet using dichloromethane, petroleum ether, diethyl ether, and n-hexane. Oracz et al. [37] reported that among the tocopherols identified in cocoa beans from different cultivars, (γ + β)-tocopherol showed the highest concentration.

TPC removal showed a variation of 2.8 times between the samples obtained, with the highest values observed at 70 °C and 60 min (runs 7 and 8) using ethyl acetate, which provided ~72% greater recovery compared to the sample obtained from n-hexane extraction. This is explained by the fact that polar solvents are more efficient in extracting phenols, as evidenced by Khursheed et al. [38]. It was also found that increasing the time and solvent:sample ratio variables resulted in greater recovery of these compounds at 70 °C, when comparing runs 6–8 and 7–8, respectively. The increase in temperature, from 40 to 70 °C, stands out in TPC removal, with increases of 1.49 and 2.61 times with solvent:sample ratios of 8 and 12, respectively, at 60 min. The increase in the solubility of gallic acid up to 60 °C is reported by Daneshfar et al. [39].

Regarding the oxidative state of the lipid fraction, the lowest value of conjugated dienes was obtained for the condition with the highest removal of TPC and total compounds (run 8). However, in general, the low values obtained (>2.77 mmol/L) indicate a low degree of primary oxidation, which may be attributed to the fact that cocoa husk fat is composed mainly of saturated fatty acids and has low levels of polyunsaturated fatty acids [5,6]. Bahramian et al. [18] obtained conjugated dienes values of 4.97 to 8.31 (mmol/L) for flaxseed oil obtained from the UAE with n-hexane and attribute the high values to the oil’s composition being rich in unsaturated fatty acids, which are partially oxidized during the ultrasonic process due to the heat generated.

**Figure 2:**
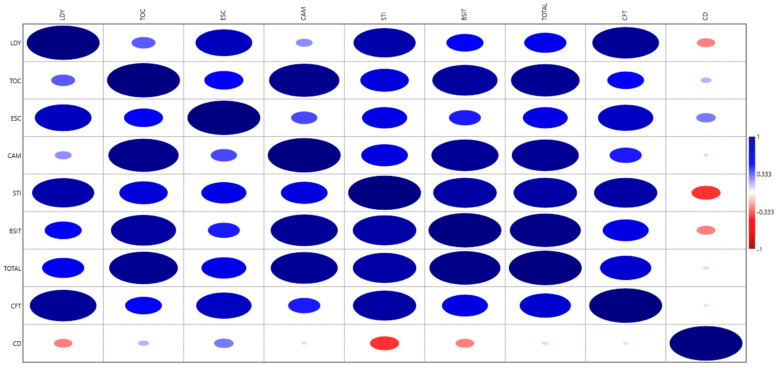
Correlation matrix describing the relationship between lipid fraction yield (LFY), y-tocopherol (TOC), Escalene (ESC), Campesterol (CAM), Stigmaterol (STI), Total compounds (Total), total phenolic compounds (TPC) and conjugated dienes (CD). The size of the ellipse is proportional to Pearson’s correlation coefficient.

Figure 2 presents the Pearson correlation matrix for the data presented in Table 4. LFY showed positive correlations with extracted compounds, especially with STI (r = 0.850), ESC (r = 0.776), CAM (r = 0.627), and TPC (0.914), suggesting that extraction efficiency is associated with the co-extraction of lipophilic and phenolic compounds. In general, a strong clustering was observed among the minor compounds, with high positive correlation coefficients, highlighting the relationships between TOC and CAM (r = 0.964), TOTAL and BSIT (r = 0.973), TOTAL and CAM (r = 0.918), TOTAL and STI (r = 0.871), and BSIT and CAM (r = 0.920). This behavior indicates strong collinearity among lipophilic constituents, suggesting co-extraction and physicochemical similarity within the analyzed fraction.

CFT also showed relevant correlations with lipophilic variables, highlighting the associations with STI (r = 0.866), TOTAL (r = 0.700), and BSIT (r = 0.629), in addition to a moderate correlation with TOC (r = 0.501). These results suggest an interaction between phenolic and lipophilic compounds, since the literature demonstrates that phenolics can act in conjunction with tocopherols in the stabilization of lipid systems through redox regeneration mechanisms and disruption of radical chains [40].

CD values showed predominantly negative correlations with the other properties evaluated, especially STI (r = −0.395), CFT (r = −0.070), TOTAL (r = −0.086), and CAM (r = 0.070). Although of low magnitude, this pattern indicates an inverse trend between the recovery of these compounds and the formation of primary lipid oxidation products, and that phenolic and lipophilic compounds.

### 3.3. Fatty Acid Profile

Table 5 presents the fatty acid composition of the lipid fraction obtained with ethyl acetate and n-hexane as extraction solvents. The fatty acid profile was not influenced by the solvent (*p* > 0.05), and the distribution between saturated, monounsaturated, and polyunsaturated fatty acids shows a lipid profile characteristic of cocoa derivatives [33,36]. The study found a predominance of saturated fatty acids (palmitic and stearic), which are directly associated with greater stability of the oil. Polyunsaturated fatty acids were found in low concentrations, which contributes to the oil’s greater resistance to primary oxidation, consistent with the low values of conjugated dienes observed in Table 4. Despite recent recommendations for the general population to avoid consuming saturated fatty acids, the nutritional impact of these fatty acids is strictly related to the balance between them and other macronutrients [41].

### 3.4. Thermodynamic Analysis of Solubility

Table 6 presents the solubility results calculated for each solute in ethyl acetate and n-hexane at 298.15 K (25 °C), after applying methodological corrections. The activity coefficients (γ) were obtained from the COSMO-SAC engine.

The results demonstrate the importance of the implemented corrections. For γ-tocopherol and squalene, both liquids at 298.15 K, the prediction of complete miscibility (*X_i_* = 1) is consistent with their physical properties. The reported solubilities for these compounds (2.28 and 2.09 mol/L, respectively) represent their pure molar densities, indicating that the solvent can accommodate the solute in any proportion.

For the solid phytosterols (campesterol, β-sitosterol, and stigmasterol), the calculated solubilities range from 0.10 to 0.22 mol/L in n-hexane and 0.12 to 0.57 mol/L in ethyl acetate. These values are physically consistent with the limited solubility behavior of sterols in organic solvents, with higher solubility observed in ethyl acetate due to its moderate polarity and greater capacity to stabilize intermolecular interactions. Proper consideration of the $\Delta G_{fus}$ and the molar fraction limitation was crucial to achieving these realistic results.

Gallic acid, a polar compound with a high $\Delta G_{fus}$ and a high activity coefficient (γ = 10) in n-hexane, exhibits the lowest solubility (0.0052 mol/L). This reflects the low affinity between the polar solute and the nonpolar solvent, as well as the strong crystalline network of gallic acid. Ethyl acetate, on the other hand, showed ~4 times greater solubility (0.0216 mol/L), demonstrating the higher intermolecular affinity between the solute and the solvent. The results obtained highlight three distinct thermodynamic regimes:

1. Complete Miscibility Regime: Liquid compounds such as γ-tocopherol and squalene exhibit $\Delta G_{fus}$ = 0. The model predicts the absence of a thermodynamic limitation associated with melting for γ-tocopherol, allowing for high molar fractions in solvents [27,28].
2. Energy-Controlled Fusion Regime: Phytosterols such as campesterol, β-sitosterol, and stigmasterol exhibit high free fusion energies, associated with stable crystalline structures [29]. Despite favorable interactions with the nonpolar solvent, solubility is mainly limited by the energy penalty of fusion.
3. Thermodynamic Incompatibility Regime: Gallic acid exhibits high polarity and a strong crystal lattice, resulting in high γ and ΔG_fus_ values, which lead to extremely low solubility in nonpolar solvents [30].

Solubility results from the competition between the free energy of fusion (ΔG_fus_) and non-ideality of the solution (γ). This relationship is consistent with classical descriptions of solution thermodynamics [26].

Based on the calculated sigma profiles of the compounds in the two solvents (Figure S1, Supplementary Materials), it can be observed that, although ethyl acetate and n-hexane have significantly different dielectric constants, the larger molecular compounds, such as campesterol, stigmasterol, and β-sitosterol, exhibit a predominantly hydrophobic core, only one hydroxyl group as the main polar site, and low electronic rearrangement in response to the medium. As a consequence, the additional polarization induced by the solvent is relatively small, resulting in quite similar sigma profiles between the different media evaluated.

Thus, it can be inferred that the observed differences in the calculated solubilities stem mainly from the different intermolecular interactions between the sigma profiles of the solutes and solvents, especially due to the distinct electronic characteristics of ethyl acetate and n-hexane in the regions associated with the electrostatic and hydrogen bonding contributions predicted by the COSMO-SAC model. Small differences in the polar regions of the sigma profiles can produce significant variations in the activity coefficients, due to the exponential nature of the residual contribution in the COSMO-SAC formalism. Regarding the values of the compounds obtained in the extractions, the discrepancy between the theoretical thermodynamic solubility (where γ-tocopherol appears as miscible in n-hexane, $X_{i}$ = 1.0, and the experimental extraction result (not detected) is a common phenomenon in natural product chemistry and can be explained by four main factors related to the solid matrix:

1. Solubility versus Extractability: The COSMO-SAC calculation predicts the equilibrium solubility between the pure solute and the solvent. However, in a solid matrix extraction (such as cocoa husks), the process depends not only on the solvent-solute affinity but also on the solvent’s ability to break solute-matrix interactions. γ-Tocopherol has a phenolic hydroxyl group that may form strong hydrogen bonds with polar components of the husk (such as lignin or carbohydrates). n-Hexane, being a pure nonpolar solvent, does not have the strength to break these interactions, while this is possible with the use of ethyl acetate (more polar).
2. Matrix Effect and Accessibility (Encapsulation): The compounds in cocoa husks are not “free-floating”; they may be located within cellular structures or hydrophilic compartments. n-Hexane has a low capacity to “wet” and penetrate polar or hydrated regions of the plant matrix. If γ-tocopherol is confined to these regions, n-hexane simply does not come into contact with it, resulting in zero extraction, regardless of how soluble the compound would be if it were free.
3. Mass Transfer Kinetics: Extraction is a diffusion-governed process. If the resistance to mass transfer within the shell pore is too high for n-hexane (due to its viscosity or lack of affinity with the diffusion channel), the extraction time may not have been sufficient for any detectable quantity to exit the matrix, even if the equilibrium favored dissolution.
4. Co-solutes and Selectivity: Squalene was well extracted by n-hexane (28.19 mg/100 g of husk). This occurs because squalene is a pure hydrocarbon, without polar functional groups, facilitating its release. γ-tocopherol and campesterol, on the other hand, possess hydroxyl groups (-OH). This small difference in polarity may be the “watershed” that prevents n-hexane from removing them from the solid matrix, acting as a solvent that is too selective for this specific application.

**Figure 3:**
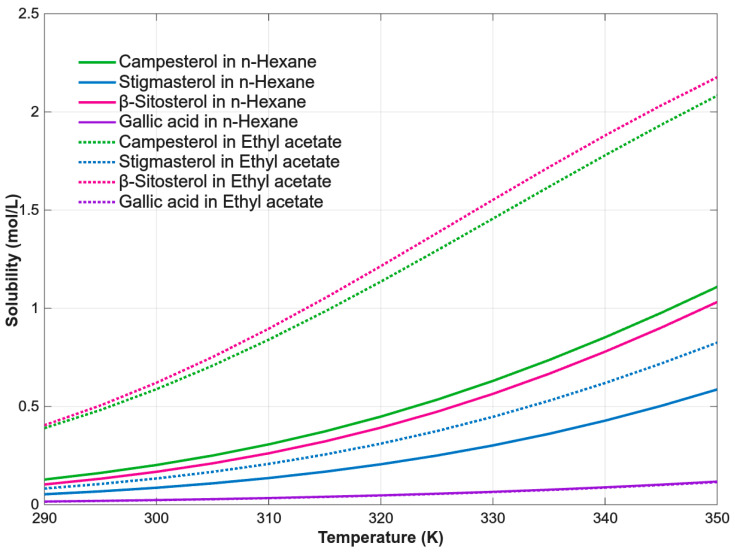
Calculated solubility variation in solid solutes in *n*-hexane and ethyl acetate over the studied temperature range.

Although the discussion above was primarily based on calculations performed at 298.15 K to facilitate comparison with literature data, the temperature-dependent solubility calculations for the solid solutes demonstrated a progressive increase in solubility with increasing temperature (Figure 3). The non-linear behavior observed for the phytosterols is consistent with the exponential temperature dependence of the solid–liquid equilibrium term, indicating that the energetic penalty associated with fusion becomes progressively less restrictive at higher temperatures.

The divergence between ethyl acetate and n-hexane also becomes more pronounced with increasing temperature, particularly for campesterol and β-sitosterol. This behavior suggests that once the fusion energy penalty is partially overcome, solvent–solute intermolecular affinity becomes the dominant factor controlling solubility. Ethyl acetate, therefore, benefits more strongly from the temperature increase due to its greater capacity to stabilize polar interactions.

In contrast, gallic acid exhibited only a modest increase in solubility with temperature, indicating that thermodynamic incompatibility remains the dominant limiting factor even at elevated temperatures.

Overall, the temperature-dependent calculations indicate that increasing temperature substantially enhances the solubility of phytosterols, particularly in ethyl acetate. However, the persistence of low experimental extraction yields for some compounds suggests that matrix-related effects, such as restricted accessibility, strong solute–matrix interactions, and mass transfer limitations, may play a more decisive role in the extraction process than equilibrium thermodynamic solubility alone.

## 4. Conclusions

The efficient removal of the lipid fraction from cocoa husks using ethyl acetate was reported in this study, considering yield and quality. Temperature and time are the variables with the greatest influence on the extraction process, providing higher LPY at 70 °C and 60 min, as well as greater recovery of minor compounds and total phenolics under these experimental conditions. Higher levels of minor compounds and total phenolics were obtained when compared to the use of n-hexane (in the selected experimental condition), and consequently, a lower value of conjugated dienes was observed. The fatty acid profile of the lipid fractions obtained indicates a predominance of palmitic and stearic acids and was not influenced by the extraction solvent. Analysis of thermodynamic solubility calculations via COSMO-SAC demonstrated that the model accurately describes the solubility regimes and the non-ideality of the liquid and solid phases of the studied compounds. However, for the purposes of designing plant extraction processes, computational thermodynamic equilibrium must be interpreted in conjunction with matrix effects and mass transfer limitations, which act as kinetic barriers in the recovery of natural compounds. The findings of this research highlight the relevance of the selected extraction and solvent method, providing data not previously available and encompassing a greater number of characteristics of the lipid fraction compared to previous work. For future work, other emerging extractive methods can be applied to compare the characteristics of the lipid fraction obtained in this study. Additionally, the applicability of the obtained LP should be investigated, especially regarding the content of non-lipid compounds present in its composition. These compounds may act beneficially in the use of LP, for example, in the formulation of organogels, which can act as a structuring agent (a hypothesis to be tested).

## Figures and Tables

**Table 1:** Thermodynamic input data for solubility calculations.

Compound	Molar Mass (g/mol)	Density (g/mL)	*T_m_* (K)	$\Delta H_{fus}$ (kJ/mol)	Note/Reference
n-hexane	86.18	0.655	na	na	Default properties [27]
Ethyl acetate	88.11	0.902	na	na	
γ-Tocopherol	416.7	0.95	na	na	Treated as a liquid (miscible) at 298.15 K [28]
Campesterol	400.7	1.05	430.65	34.5	Average experimental value for C28 phytosterols [29]
β-Sitosterol	414.7	1.05	413.15	36.5	Adjusted according to Differential Scanning Calorimetry data [29]
Stigmasterol	412.7	1.05	443.15	36.0	Classic value for C29 unsaturated sterols [29]
Squalene	410.7	0.858	~198	28.0	Melting point below 298.15 K, behaving like a liquid [27]
Gallic acid	170.12	1.69	533.15	28.0	Physicochemical data [27,28]; $\Delta H_{fus}$ estimated by thermodynamic analogy with similar aromatic phenolics [26,30]

na: not applicable (treated as liquids). ΔH_fus_ of gallic acid was estimated by molecular analogy using the Yalkowsky group contribution method [30] for functionalized aromatic phenolic compounds and subsequently adjusted to ensure phase consistency in the COSMO-SAC predictive model according to the classical ideal solubility equilibrium criteria [28].

**Table 2:** Experimental conditions and lipid fraction yield (LFY) results.

Run	Variable			LFY (wt%)
	T (°C)	t (min)	R (mL/g)	
1	−1 (40)	−1 (20)	−1 (8)	7.12
2	−1 (40)	−1 (20)	1 (12)	9.97
3	−1 (40)	1 (60)	−1 (8)	8.40
4	−1 (40)	1 (60)	1 (12)	11.18
5	1 (70)	−1 (20)	−1 (8)	7.44
6	1 (70)	−1 (20)	1 (12)	8.44
7	1 (70)	1 (60)	−1 (8)	9.38
8	1 (70)	1 (60)	1 (12)	12.97 ± 0.28 ^1^
9–12	0 (55)	0 (40)	0 (10)	8.09 ± 0.10 ^1^

T: temperature; t: extraction time; R: solvent/sample ratio. ^1^ experiments conducted in quadruplicate.

**Table 3:** Effect of independent variables on lipid fraction yield.

Variable	Effect	*p*-Value
Mean/Interaction	8.96	<0.001
T	2.64	<0.001
t	2.32	<0.001
R	0.47	<0.01
T × t	0.71	<0.001
T × R	−0.18	<0.200
t × R	1.08	<0.001
T × t × R	0.74	<0.001

T: temperature; t: extraction time; R: solvent/sample ratio.

**Table 4:** Lipid fraction yield (LFY), contents of minor compounds and total phenolic compounds (TPC) removed from the husks for the lipid fraction, and value of conjugated dienes (CD) in the lipid fraction.

Run ^1^	LFY (wt%)	Compound (mg/100 g Husks)						TPC (mg GAE/100 g Husks)	CD (mmol/L)
		(γ + β)-Tocopherol (TOC)	Escalene (ESC)	Campesterol (CAM)	Stigmaterol (STI)	β-Sitosterol (BSIT)	Total		
2	9.97 ± 0.05	4.01 ^a^ ± 0.1	6.43 ^a^ ± 0.4	3.32 ^a^ ± 0.3	6.97 ^a^ ± 0.1	21.27 ^a^ ± 0.2	42.01 ^a^ ± 0.3	1.22 ^a^ ± 0.1	2.40 ^c^ ± 0.02
3	8.40 ± 0.26	4.72 ^a^ ±0.1	6.21 ^a^ ± 0.2	3.39 ^a^ ± 0.3	7.40 ^ab^ ± 0.5	21.27 ^a^ ± 0.9	42.99 ^a^ ± 0.8	1.79 ^b^ ± 0.1	2.56 ^c^ ± 0.01
4	11.18 ± 0.42	5.98 ^b^ ± 0.1	7.73 ^b^ ± 0.2	3.95 ^ab^ ± 0.1	8.48 ^b^ ± 0.2	25.76 ^b^ ± 0.3	51.90 ^d^ ± 0.6	1.34 ^a^ ± 0.1	2.27 ^b^ ± 0.16
6	8.44 ± 0.17	5.52 ^b^ ± 0.2	7.53 ^b^ ± 0.1	3.17 ^a^ ± 0.3	7.74 ^ab^ ± 0.5	22.56 ^a^ ± 0.3	46.53 ^c^ ± 0.7	1.37 ^a^ ± 0.1	2.25 ^b^ ± 0.01
7	9.38 ± 0.14	7.54 ^c^ ± 0.1	12.27 ^c^ ± 0.6	4.15 ^ab^ ± 0.1	8.72 ^b^ ± 0.5	24.39 ^b^ ± 0.7	57.07 ^e^ ± 0.1	2.68 ^c^ ± 0.1	2.77 ^d^ ± 0.03
8	12.97 ± 0.28	8.50 ^c^ ± 0.1	10.11 ^c^ ± 0.4	5.62 ^b^ ± 1.1	11.7 ^c^ ± 0.6	35.20 ^c^ ± 0.8	71.17 ^f^ ± 1.5	3.50 ^d^ ± 0.4	2.08 ^a^ ± 0.09
*n*-hexane	10.94 ± 0.34	nd	8.06 ^b^ ± 0.6	nd	7.69 ^ab^ ± 0.1	14.59 ^d^ ± 0.1	30.34 ^b^ ± 0.4	1.98 ^ab^ ± 0.1	2.21 ^b^ ± 0.09

^1^ as Table 2. GAE: Gallic Acid Equivalents. nd: not detected. Means followed by the same lowercase letters (for the same column) indicate that there is no significant difference (*p* > 0.05).

**Table 5:** Fatty acid composition of the lipid fraction obtained with ethyl acetate and n-hexane as extraction solvent.

Fatty Acid (%) ^1^	Ethyl Acetate	*n*-Hexane
Palmitic	28.99 ^a^ ± 0.12	30.28 ^a^ ± 0.27
Stearic	33.78 ^a^ ± 0.10	31.73 ^a^ ± 0.17
Oleic	32.94 ^a^ ± 0.42	33.82 ^a^ ± 0.15
Linoleic	2.76 ^a^ ± 0.01	2.86 ^a^ ± 0.07
Arachidic	1.53 ^a^ ± 0.22	1.11 ^a^ ± 0.02
Sum of saturated	64.30 ^a^ ± 0.27	63.12 ^a^ ± 0.32
Sum of unsaturated	35.70 ^a^ ± 0.42	36.68 ^a^ ± 0.17
Sum of polyunsaturated	2.76 ^a^ ± 0.01	2.86 ^a^ ± 0.07

^1^ Percentage of the normative area. Means followed by the same letters (in each line) do not show a significant difference (*p* > 0.05).

**Table 6:** Calculated thermodynamic properties for different solutes in ethyl acetate and n-hexane media.

Solute	γ ^a^	$\Delta G_{fus}$ (kJ/mol)	Molar Fraction ($X_{i}$)	Solubility (mol/L)
**Ethyl acetate**				
γ-tocopherol	0.7346	0.0000 ^b^	1.0000 ^c^	2.2798 ^d^
Campesterol	0.2181	10.6148	0.0633	0.5474
β-Sitosterol	0.2442	10.1597	0.0680	0.5765
Stigmasterol	0.6964	11.7793	0.0124	0.1223
Squalene	0.1500	0.0000 ^b^	1.0000 ^c^	2.0891 ^d^
Gallic acid	3.2639	12.3417	0.0021	0.0216
***n-*** **hexane**				
γ-tocopherol	0.8504	0.0000 ^b^	1.0000 ^c^	2.2798 ^d^
Campesterol	0.5376	10.6148	0.0257	0.1862
β-Sitosterol	0.5500	10.1597	0.0302	0.2163
Stigmasterol	0.6000	11.7793	0.0144	0.1063
Squalene	1.0000	0.0000 ^b^	1.0000 ^c^	2.0891 ^d^
Gallic acid	10.0000	12.3417	0.0007	0.0052

^a^ Activity coefficients (γ) obtained by COSMO-SAC. ^b^ ΔG_fus_ = 0 kJ/mol indicates that the pure solute is in the liquid state at 298.15 K. ^c^ X_i_ = 1 reflects the complete miscibility regime achieved due to the absence of a fusion barrier combined with the high thermodynamic affinity (γ) observed in the system. ^d^ Solubility values for the completely miscible compounds (X_i_ = 1) correspond to their respective pure molar densities at 298.15 K.

## Data Availability

The original contributions presented in this study are included in the article/Supplementary Materials. Further inquiries can be directed to the corresponding author.

## Supplementary Materials

The following supporting information can be downloaded at https://www.mdpi.com/article/10.3390/foods15132275/s1. Table S1. Preliminary test comparing extraction conducted in one and two stages and solvents. Table S2. Analysis of variance of the results obtained for lipid fraction yield (Table 1). Figure S1. Calculated σ-profiles at the BP86/def2-SVP level of theory for the investigated solutes in ethyl acetate (left) and n-hexane (right) media.
