# Supplementary material for: Separation of the Lipid Fraction from Cocoa Bean Husks Using Ethyl Acetate as Solvent in Ultrasound-Assisted Process
Source: Foods. 2026 Jun 25;15(13):2275. doi: 10.3390/foods15132275 (PMC13361922; doi:10.3390/foods15132275)
Supplement: Supplementary file 1 [file foods-15-02275-s001.zip › foods-4359881-supplementary.pdf]

Supplementary Materials

Table S1. Preliminary test comparing extraction conducted in one and two stages and solvents.

Solvent	Lipid fraction yield (wt%)	
	1º step	2ª step
Ethyl acetate	7.19 ± 0.19	8.42 ± 0.28
Ethanol	6.81 ± 0.13	7.43 ± 0.10

Extraction conducted at 60 °C, 60 min e solvente/sample ratio of 12 mL/g.

Table S2. Analysis of variance of the results obtained for lipid fraction yield (Table 1).

	Sum of squares	Degrees of freedom	Medium square	$F_{calculated}$	$F_{critical}$
Regression	66.22	6	11.04		
Residue	4.73	8	0.59		
Lack of it	4.47	2	2.24	18.66	3.58
Pure error	0.26	6	0.04		
Total	70.69	14	5.05		

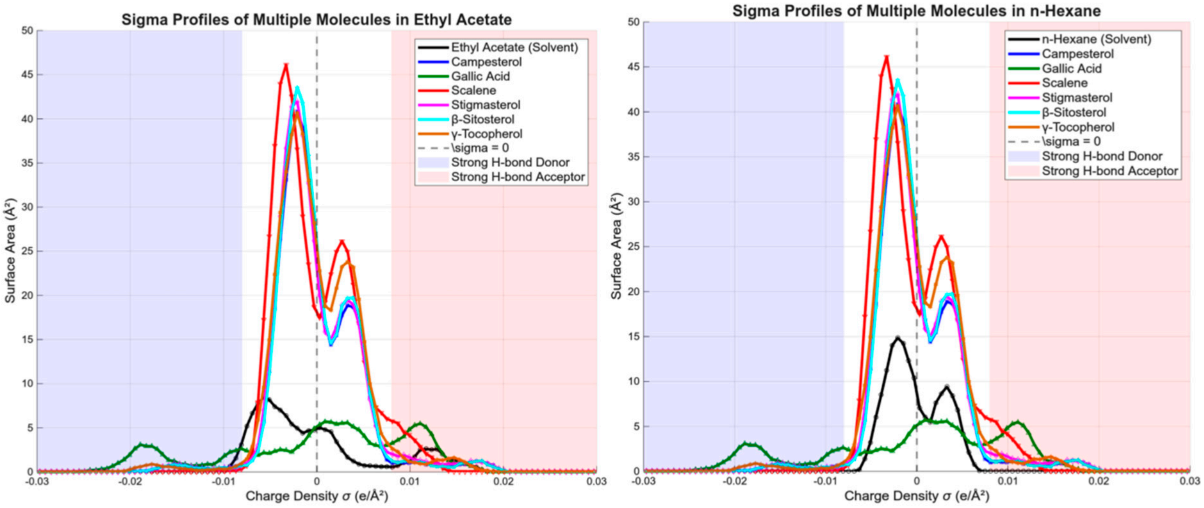

Figure S1. Calculated σ-profiles at the BP86/def2-SVP level of theory for the investigated solutes in ethyl acetate (left) and n-hexane (right) media. The shaded areas represent the regions for strong hydrogen-bond donors (blue region, $\sigma < -0.0084$ e/Å^2) and strong hydrogen-bond acceptors (red region, $\sigma > 0.0084$e/Å^2). The central dashed line ($\sigma = 0$) highlights the nonpolar/hydrophobic region.
